# Principal Component Analysis of the Well-Being at Work and Respect for Human Rights Questionnaire (WWRRR) in the Mediterranean Region

**DOI:** 10.2174/1745017902016010115

**Published:** 2020-07-30

**Authors:** Mathilde Husky, Yosra Zgueb, Uta Ouali, Cesar I. A. Gonzalez, Martina Piras, Giorgia Testa, Alberto Maleci, Alfredo Mulas, Alessandro Montisci, Samih Nujedat, Goce Kalcev, Iskren Teodorov, Antonio Preti, Matthias Angermeyer, Mauro Giovanni Carta

**Affiliations:** 1 Laboratoire de Psychologie, Université de Bordeaux, Bordeaux, France; 2 Department of Psychiatry A, Razi Hospital, La Manouba, Tunisia; 3 Faculty of Medicine of Tunis, University of Tunis El Manar, Tunis, Tunisia; 4 Department of Medical Sciences and Public Health, University of Cagliari Cagliari, Italy; 5 Innovation Sciences and Technologies, University of Cagliari, Cagliari, Italy; 6 Department of Medical Sciences and Public Health, University of Cagliari, Cagliari, Italy; 7 Center for Public Mental Health, Gosim Austria

**Keywords:** Questionnaire, Principal component, Human rights, Job satisfaction, Wellbeing, Mental health, Mediterranean area

## Abstract

**Background:**

The Well-Being at Work and Respect for human Rights Questionnaire (WWRR) was conceived based on the hypothesis that the perception of respect for users' rights is an essential element of well-being in the workplace in healthcare. The objective of the study is to examine the principal components of the WWRR.

**Methods:**

A random sample representative of a set of professionals working in three different healthcare networks in Tunisia, North-Macedonia, and Italy was enrolled (n=426). Each professional completed a questionnaire on sociodemographic data and the WWRR. The WWRR consists of six items on beliefs about: satisfaction at work, users’ satisfaction, organization at work, respect of users’ and staff human rights, adequacy of resources. A seventh item assesses the perceived needs of personnel. Correlation between the items was evaluated by analysing the principal components with Varimax rotation and Kaiser normalization (which included all components with an Eigen value> 1).

**Results:**

A single factor covered over 50% of the variance, all the items of the questionnaire were closely related and compose a single factor. Tunisia presented some differences regarding the item about the human rights of staff.

**Conclusion:**

Satisfaction with the respect for the rights of users is strongly correlated with the other factors that are part of the concept of the organizational well-being of health care providers. The WWRR provides a means of measuring this important and often neglected dimension.

## 1 INTRODUCTION

The United Nations Convention on the Rights of Persons with Disabilities [1] emphasized the importance of respecting the rights of persons who are in need of receiving care and treatment. In the current conception of care systems, the perception of respect for users' rights may be therefore an essential element of general well-being, both for users and for the healthcare personnel providing the care. The Questionnaire on Well-Being at Work and Respect for Human Rights (WWRR) was conceived and structured based on these concepts and on the hypothesis that the perception of respect for users' rights is an essential element of well-being in the workplace in healthcare. The instrument further integrates other components of the construct of well-being in the workplace previously described in the literature and which are considered determinants of organizational well-being and work-related stress [2, 3]. The perception of respect for users' rights is even more important in sectors such as mental health in which the issue over the non-respect of rights is particularly relevant [4, 5].

The objective of the study is to examine the factorial structure of the Well-Being at Work and Respect for Human Rights Questionnaire administered in three different Mediterranean contexts.

## 2 METHODS

### 2.1 Design

A pilot study was conducted in mental health care facilities in three Mediterranean countries: Tunisia, Macedonia, and Italy. These three countries were selected based on their diverse cultural, socioeconomic, and religious contexts. In each country, different mental health facilities were selected for participation.

In Tunisia, the study was conducted at Razi University Hospital, the only hospital dedicated to the treatment of mental illness in Tunisia. It is situated in the Greater Tunis area and has a total number of seven adult inpatient services, one adult outpatient and emergency department, one department of child and adolescent psychiatry, and one department of forensic psychiatry. In this study, staff in non-psychiatric services including the Laboratory and Pharmacy department and in the administration were enrolled.

In North Macedonia interviews were conducted on the staff of psychiatric units and outpatient mental health facilities. In addition, staff from counseling and psychotherapy units and the Private Units for Mental Diseases were offered participation.

In Sardinia, Italy, the study was conducted in the mental health community services and in the hospital wards for psychiatric emergencies (in Italy there are no psychiatric hospitals) of the Department of Mental Health ASL8. This is a community network that provides care for over 500.000 adults.

The city of Cagliari and its vicinity consist of six units of territorial care and two hospital wards for emergencies at the general hospital of 15 beds each.

### 2.2 Sample

In each facility, a random sample representative of the set of professionals working in mental health was contacted by email and offered participation, which consisted of completing a 10-minute questionnaire. Depending on the country, a target sample of 250 participants was pursued. All participants provided written informed consent. The response rate varied by country, with an overall response rate of 68.9% [from 66.2% Tunisia to 84.4% in North-Macedonia]. As per the protocol of the study, for the reason of privacy, no information was recorded about those who did not accept to take part in the survey, since they did not return the signed informed consent.

The final sample included a total of 426 health workers. The sample characteristics are presented in Table **1**.

### 2.3 Instruments

#### 2.3.1 Socio-demographic variables

In each setting, sociodemographic questionnaires collected data on the following variables: Age, Gender, Occupational Status, Education, Working Shift, Type of Working Contract, Place of employment.

#### 2.3.2 Well-Being at work and respect for human rights questionnaire (WWRR).

The scale is part of a global World Health Organization initiative on human rights and the implementation of the Convention on the Rights of Persons with Disabilities, the Quality Rights initiative (https://www.who.int/ mental_health/policy/quality_rights/en/). The main aims of the scale are to measure how the respect of the human rights of patients and staff is perceived by patients and staff and how this is linked to organizational and working climate. The questionnaire was developed based on discussions with different professionals including psychiatrists, psychologists, rehabilitation technicians, and psychometrists. The goal was to pilot a short, simple, and easy to use tool for future use in large multi-center studies. To date, the WWRR has been translated and back-translated from Italian to English, French, Macedonian, and Maghreb Arabic. The core items of the questionnaire are the first six. The seventh item is exploratory in nature as the perception of the need for resources of different types of personnel and/or teams may be informative. This is the first validation study of this instrument, exploring its factorial structure. Furthermore, the scale is under validation across 24 countries that are part of the Quality Rights initiative. The questionnaire is intended for the use of health care professionals in different health care settings.

The items of the WWRR are the following:

(1) How satisfied are you with your work? (Likert scale from 1 Not at all to 6 Completely satisfied)

(2) How much you believe that the users of the service in which you work are satisfied? (Likert scale from 1 Not at all to 6 Completely satisfied)

(3) How satisfied are you with the organizational aspects of your work /how your work is organized? (Likert scale from 1 Not at all to 6 Completely satisfied).

(4) To what extent do you believe that the human rights of the people who are cared for in your service are respected? (Likert scale from 1 Not at all to 6 completely respected)

(5) To what extent do you believe that the human rights of the staff working in your service are respected? (Likert scale from 1 Not at all to 6 completely respected)

(6) How do you evaluate the current state of care in mental health in your service/ward, with reference to resources? (1- the resources are adequate; 2 - would like to have more resources but those present are however quite congruous; 3 - There are defects but it is possible to provide sufficiently valid assistance; 4 - Resources are insufficient and inadequate assistance is provided; 5 - Poor assistance is provided due to serious resource deficits)

(7) Which types of professionals do you think would be most useful to add in your service (only one possible answer): Doctors, Psychologists, Nurses, Educators or Rehabilitation Technicians, Social Assistants, support staff, security personnel.

### 2.4 Statistics

First, sample characteristics were examined using chi-square and t-tests. Second, several principal component analyses with Varimax rotation and Kaiser normalization (which included the inclusion of all components with an Eigen value> 1) were conducted on the first six items of the questionnaire. Data were analysed with the SPSS-SP 23.0 software package (SPSS Inc., Chicago Illinois).

### 2.5 Ethical Considerations

The research was approved by the ethics committee of the Azienda Mista Ospedaliero Universitaria di Cagliari and was conducted in accordance with the Helsinki declaration of ethical principles.

## 3 RESULTS

### 3.1 Sample Characteristics

Sample characteristics are presented in Table **1**. No relevant differences were shown by gender between the three samples. The Italian sample was significantly older than the sample in the two other countries, [See **Table 1**: Italy as Pivot (dichotomized <40 *vs*> 39), *vs* Macedonia (χ^2^ = 61.864, df=1, p <0.0001) *vs* Tunisia (χ^2^ = 147.15, df=1, p <0.0001)]. The Macedonian sample held a greater number of doctors than the other two countries [See Table **1**: Medical Doctors *vs* others professionals - Italy as Pivot, *vs* Macedonia (χ^2^= 14.21, 1 df=1, p <0.0001); *vs* Tunisia (χ^2^ = 1.498, df=1, p = 0.22)], The Macedonian sample also showed a greater number of psychologists as compared to the Tunisian sample [Italy as Pivot - *vs* Macedonia (χ^2^= 2.927,df=1, p = 0.08); *vs* Tunisia (χ^2^= 2.126, df=1, p = 0.14); given the divergent figure Macedonia *vs* Tunisia (χ^2^= 11.238, df=1, p = 0.001)].

In the Italian sample, a larger proportion of staff workers had permanent employment due to the presence of a fixed-term work contract in all the workers interviewed [(Full Time *vs* Part) - Italy pivot - to Macedonia (χ^2^= 670.84, df=1, p <0.0001) to Tunisia (χ^2^ = 160.48, df=1, p <0.0001)]. The place of work in Italy is more frequent in community care services, in Tunisia in Psychiatric Hospital.

### 3.2 Principal Components Analysis in the Total Sample

A single factor covers over 50% of the variance (Table **2**). The component matrix illustrates how all the items of the questionnaire are closely related and compose a single factor, except for item 6 which is inversely correlated, as expected (Table **2**). The item on the human rights of users is strongly associated with other items while the strength of the association with the item on the human rights of staff is weaker.

**Fig. (A1):**
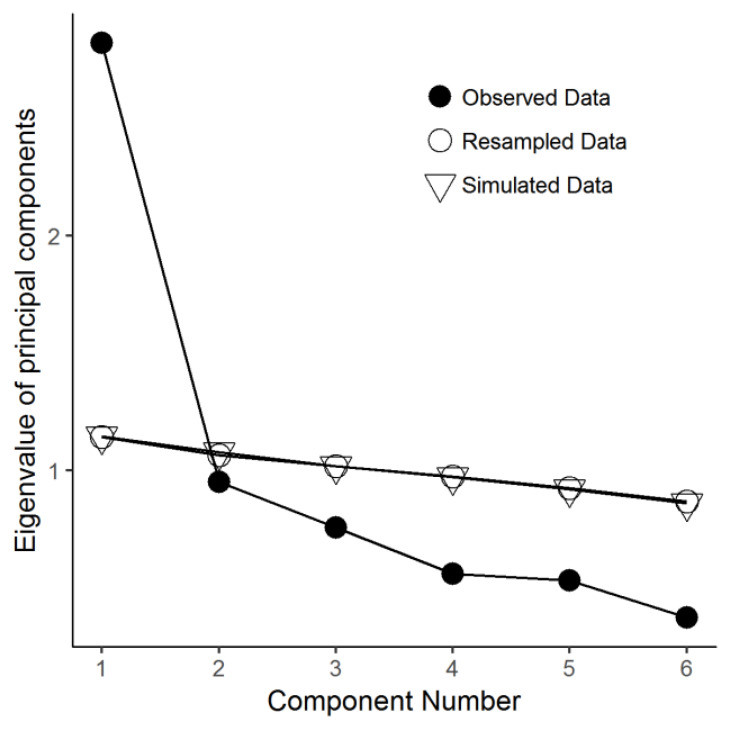
Scree plot with added parallel analysis in the whole sample (n = 426). A unidimensional solution is suggested on the basis of the Kaiser normalization and the parallel analysis.

The scree plot with added parallel analysis for the whole sample is depicted in the Appendix. (Fig. **A1** in the appendix).

### 3.3 Principal Components Analysis Within Each Country

Table **3** shows how the association of the six items is even stronger in the Italian sub-sample reflecting a strong coherence of response patterns.

**Fig. (A2):**
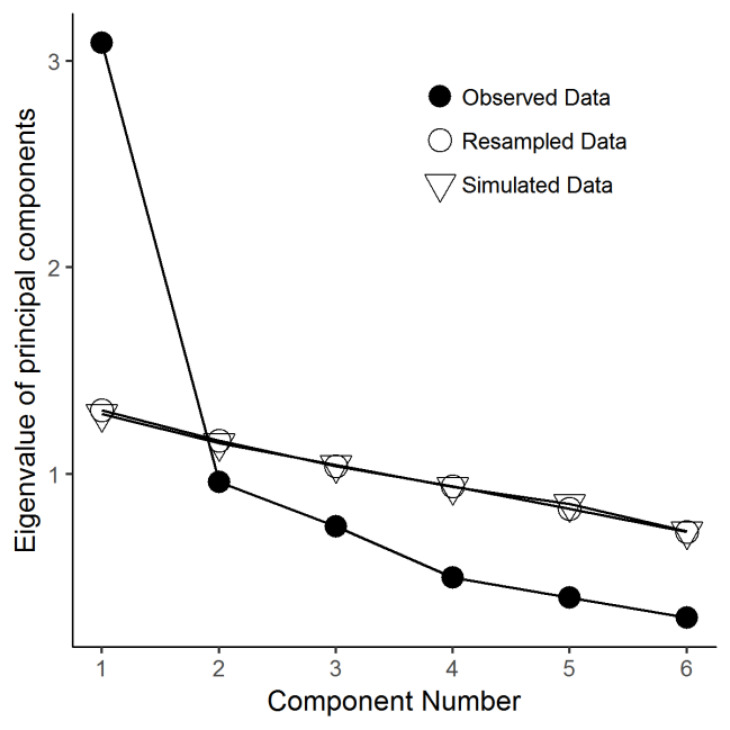
Figure A 2. Scree plot with added parallel analysis in the Italian sample (n = 126). A unidimensional solution is suggested on the basis of the Kaiser normalization and the parallel analysis.

The scree plot with added parallel analysis for the Italian sample is depicted in the Appendix (Fig. **A2** in the appendix).

The Tunisian sub-sample differs partially from the general trend with the 6-item model explaining only 36.5% of the variance. In addition, item 5 assessing beliefs about the respect of human rights of the staff does not appear to correlate with the other items and, by itself, constitutes a second factor (Table **4**).

**Fig. (A3):**
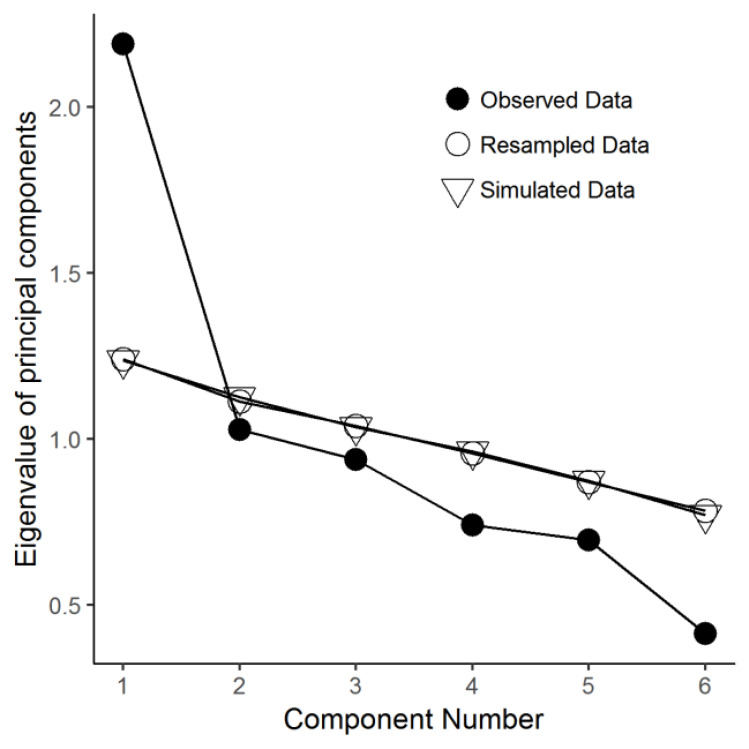
Scree plot with added parallel analysis in the Tunisian sample (n = 200). A two-factor solution is suggested on the basis of the Kaiser normalization, but the parallel analysis suggested that a unidimensional solution is acceptable.

The scree plot with added parallel analysis for the Tunisian sample is depicted in the Appendix (Fig. **A3** in the appendix).

The Macedonian sub-sample instead aligned itself with the general trend, with the six items strongly interrelated and a single factor explaining 51.5% of the variance (Table **5**).

**Fig. (A4):**
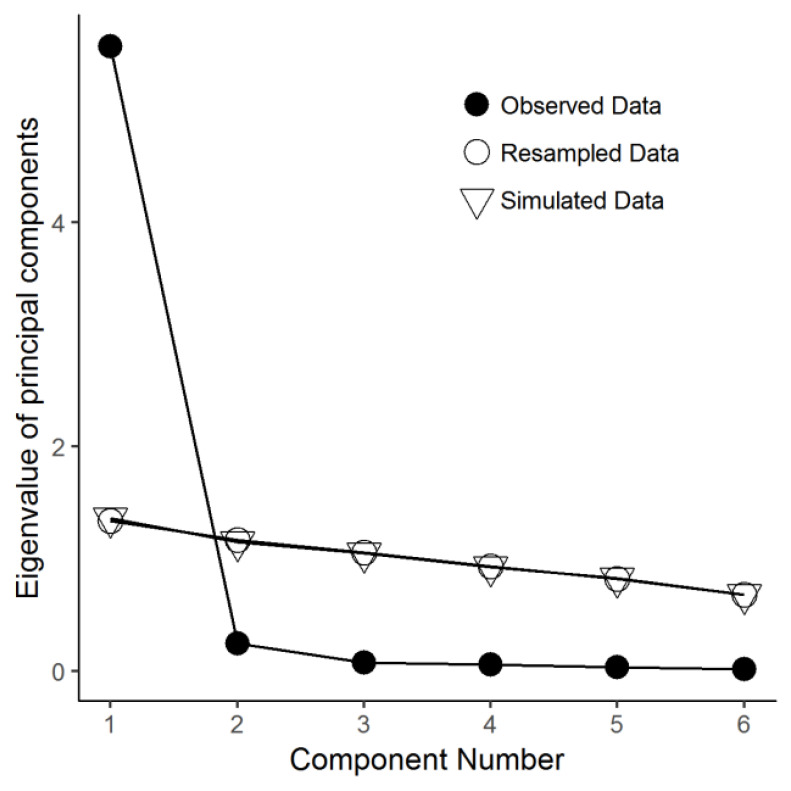
Scree plot with added parallel analysis in the North-Macedoniansample (n = 100). A unidimensional solution is suggested on the basis of the Kaiser normalization and the parallel analysis.

The scree plot with added parallel analysis for the North-Macedonian sample is depicted in the Appendix (Fig. **A4** in the appendix).

### 3.4 Principal Components Analysis Within Each Gender and Employment Stability Status

The principal components analysis carried out in the sub-samples distinguished by gender did not change the general findings although the strength of the association of the respect for staff human rights with other items was stronger among males and among females (Table **6a** and **6b**).

Similarly, analyses conducted distinguishing the sample on the basis of permanent employment status suggested the presence of a single factor (Table **7a** and **7b**).

## 4 DISCUSSION

The results of the PCA suggest that the scale measures just one main latent trait, satisfaction with work, in particular well-being at work. Furthermore, items related to the respect of human rights at work are specifically related to well-being.

The study shows that the six core items that make up the questionnaire are strongly inter-related and contribute to a single factor explaining over half of the variance. However, there are subsample variations in the manner in which the item on respect for the human rights of staff. Specifically, in the Tunisian sample and for women in the overall sample, this item does not systematically covary as strongly with other items as what was seen in Italy or Macedonia or among men in the overall sample. In fact, in Tunisia, this item stood alone in a second component, which was based on a very tiny eigenvalue (1.027) and depended on the separation of just one item (‘item 5 relative to the respect of the human rights of the staff).

In essence, the results confirm the initial hypothesis and support the concept that the perception of respect for human rights (those of users and those of workers) is a component of well-being in the workplace. The discordant result, relative to item 5, of Tunisia can be interpreted as the result of a general situation of dissatisfaction of health workers, not only of mental health. Indeed, ever since the Arab Revolution in 2011, Tunisia is suffering from a severe economic and financial crisis which also impacts the public health budget in general and the mental health budget in particular. Furthermore, the Tunisian health system has growing structural difficulties, expressed amongst others by the instability of leadership (*e.g*., during the last 8 years since the Revolution, Tunisia has had 9 Ministers of Health). Therefore, many health workers think that there is no continuity and no leadership within the ministry, resulting amongst others in feelings of hopelessness and despair concerning their professional situation. As a matter of fact, Tunisia experiences a considerable brain drain of health professionals (doctors as well as nurses) towards European countries and the Gulf States. This interpretation would also explain why the item does not correlate with others not even in the opposite direction.

Item 5 was also introduced in relation to the increase in recent literature on discrimination and prejudice against mental health workers and the controversy that arose in certain conditions in which mental health workers did not feel sufficiently protected [6].

Among women, the item regarding human rights for staff was less strongly associated with job satisfaction as it was for men. This finding may point to existing gender-based discrimination in the workplace.

While the respect for the human rights of users was consistently correlated with job satisfaction among workers, the respect for staff in the present sample yielded nuanced results. The item on human staff rights may, in fact, reflect basic democratic principles that may or may not be fully in place in various regions of the world. The fact that in Italy, the WWRR questionnaire showed more homogenous scores in the present study, can reflect aspects of its development in terms of the establishment of human rights.

Our study has the following limitations: It relied on a small sample of heterogeneous healthcare personnel using a random sampling method, and response rates could not be calculated. The present study, therefore, needs to be replicated in larger samples of healthcare workers in various cultural settings. The results of this study are applicable to the studied countries. Further studies are underway.

## CONCLUSION

Satisfaction with respect to the rights of users is strongly correlated to the other factors that are part of the concept of the organizational well-being of a healthcare provider. However, the perception of staff human rights is subject to vary as a function of gender or country. The WWRR may prove useful in expanding our understanding of the importance of the perception of respect for human rights in the field of healthcare around the world.

## Figures and Tables

**Table 1:** Sample characteristics.

		Italy	Macedonia	Tunisia	Total	Statistics
		N = 126	N = 100	N = 200	N=426	
Gender	Men Women	45 (36%) 81 (64%)	39 (39%) 61 (61%)	78 (39%) 122 (61%)	162 (38%) 264 (62%)	Italy *vs* Macedonia χ^2^ = 0.258, df =1, p = 0.61 Italy *vs* Tunisia χ^2^ = 0.0355, df=1, p = 0.55
Age	<20 years old 20-29 years old 30-39 years old 40-49 years old 50-59 years old >60 years old	0 (0%) 0 (0%) 2 (2%) 30 (24%) 74 (59%) 20 (16%)	4 (4%) 21 (21%) 19 (19%) 28 (28%) 22 (22%) 6 (6%)	1 (0.5%) 60 (30%) 79 (39.5%) 30 (15%) 29 (14.5%) 1 (0.5%)	5 (1%) 81% (19%) 100 (23.5%) 88 (20.66%) 125 (29.5%) 27 (6.34%)	Italy *vs* Macedonia dichotomized <40 vs> 39 χ^2^= 61.864, df=1, p <0.0001 Italy *vs* Tunisia dichotomized <40 vs> 39 χ^2^= 147.15, df=1, p <0.0001
Occupation	Medical Doctor Psychologist Nurse Occupational Therapist Social worker Administrative staff Security staff Other	23 (18.25%) 8(6.35%) 64 (64.8%) 8 (6.35%) 7 (5.55%) 13 (10.3%) 0 (0%) 3 (2.38%)	41(41%) 13(13%) 34 (34%) 4 (4%) 2 (2%) 6 (6%) 0(0%) 0(0%)	48(24%) 8(6.35%) 64 (50.80%) 21 (10.5%) 1 (0.5%) 32 (16%) 3 (1.5%) 0(0%)	112(26.29%) 6(3%) 187 (43.9%) 33 (7.75%) 10 (2.35%) 51 (11.98%) 3 (0.7%) 3 (0.7%)	MD *vs* others Italy *vs* Macedonia χ^2^ = 14.21, df=1, p <0.0001 Italy *vs* Tunisia χ^2^ = 1.498, df=1, p = 0.22
Kind of Work contract	Permanent Fixed T	126 (100%) 0 (%)	63 (63%) 37 (37%)	57 (28.5%) 143 (71.5%)	246 (57.75%) 180 (42.25%)	(Full Time *vs* Part) Italy *vs* Macedonia χ^2^= 670.84, df=1, p<0.0001) Italy *vs* Tunisia χ^2^ = 160.48, df=1, p <0.0001
Service	Mental health center / Outpatients service Mental hospital /psychiatric wards in general hospitals Daycare center	108 (85.71%) 12 (9.52%) 6 (4.76%)	77 (77%) 10 (10%) 13 (13%	145 (72.5%) 25 (12.5%) 30 (15%)	330 (77.46%) 47 (11.0%) 49 (11.50%)	χ^2^=9.7921, df=4,p=0.04

**Table 2:** Principal component analysis of the WWRR in the whole sample (n = 426).

	Total	% of variance	Cumulative %	Component Loading on the Factor
1. How much are you satisfied with your job?	3.05	50.8	50.8	0.706
2. How much do you think the users of your service ward are satisfied?	0.82	13.6	64.5	0.775
3. How much are you satisfied with the organizational aspect of your work /how your work is organized?	0.65	10.9	75.5	0.766
4. How much do you think the human rights of the users of your service ward are respected?	0.57	9.5	85.0	0.790
5. How much do you think the human rights of your staff are respected?	0.56	9.3	94.4	0.539
6. How do you evaluate the current state of care in mental health in your service/ward, with reference to resources?	0.33	5.5	100	-0.672
	Eigenvalues	Explained variance		
Extraction sum of the selected factor	3.05	50.8%		

**Table 3:** Principal component analysis of the WWRR in the Italian subsample (n = 126).

	Total	% of variance	Cumulative %	Component Loading on the Factor
1. How much are you satisfied with your job?	5.56	92.8	92.8	0.981
2. How much do you think the users of your service ward are satisfied?	0.24	4.1	96.9	0.972
3. How much are you satisfied with the organizational aspect of your work /how your work is organized?	0.07	1.2	98.1	0.982
4. How much do you think the human rights of the users of your service ward are respected?	0.06	1.0	99.1	0.972
5. How much do you think the human rights of your staff are respected?	0.03	0.5	99.7	0.954
6. How do you evaluate the current state of care in mental health in your service/ward, with reference to resources?	0.01	0.2	100	-0.917
	Eigenvalues	Explained variance		
Extraction sum of the selected factor	5.568	92.8%		

**Table 4:** Principal component analysis of the WWRR in the Tunisian subsample (n = 100).

	Total	% of variance	Cumulative %	Component Loading on Factor 1	Component Loading on Factor 2
1. How much are you satisfied with your job?	2.19	36.4	36.493	0.64	-0.133
2. How much do you think the users of your service ward are satisfied?	1.02	17.1	53.607	0.704	0.137
3. How much are you satisfied with the organizational aspect of your work /how your work is organized?	0.93	15.6	69.234	0.682	-0.150
4. How much do you think the human rights of the users of your service ward are respected?	0.73	12.3	81.555	0.736	0.052
5. How much do you think the human rights of your staff are respected?	0.69	11.5	93.132	-0.032	0.97
6. How do you evaluate the current state of care in mental health in your service/ward, with reference to resources?	0.41	6.8	100	-0.527	-0.158
	Eigenvalues	Explained variance			
Extraction sum of the selected factor 1	2.19	36.4%			
Extraction sum of the selected factor 2	1.02	17.1%			

**Table 5:** Principal component analysis of the WWRR in the Macedonian subsample (n = 100).

	Total	% of variance	Cumulative %	Component Loading on the Factor
1. How much are you satisfied with your job?	3.08	51.4	51.472	0.751
2. How much do you think the users of your service ward are satisfied?	0.96	16.0	67.485	0.736
3. How much are you satisfied with the organizational aspect of your work /how your work is organized?	0.74	12.4	79.913	0.808
4. How much do you think the human rights of the users of your service ward are respected?	0.49	8.3	88.229	0.740
5. How much do you think the human rights of your staff are respected?	0.40	6.7	94.945	0.788
6. How do you evaluate the current state of care in mental health in your service/ward, with reference to resources?	0.30	5.0	100	-0.400
	Eigenvalues	Explained variance		
Extraction sum of the selected factor	3.05	50.8		

**Table 6a:** Principal component analysis of the WWRRin women (n = 264).

	Total	% of variance	Cumulative %	Component Loading on the Factor
1. How much are you satisfied with your job?	3.05	50.8	50.8	0.75
2. How much do you think the users of your service ward are satisfied?	0.97	16.2	67.1	0.83
3. How much are you satisfied with the organizational aspect of your work /how your work is organized?	0.67	11.2	78.3	0.78
4. How much do you think the human rights of the users of your service ward are respected?	0.59	9.9	88.2	0.77
5. How much do you think the human rights of your staff are respected?	0.45	7.5	95.7	0.32
6. How do you evaluate the current state of care in mental health in your service/ward, with reference to resources?	0.25	4.2	100	-0.68
	Eigenvalues	Explained variance		
Extraction sum of the selected factor	3.05	50.8%		

**Table 6b:** Principal component analysis of the WWRR in men (n = 162).

	Total	% of variance	Cumulative %	Component Loading on the Factor
1. How much are you satisfied with your job?	2.94	49.0	49.035	0.65
2. How much do you think the users of your service ward are satisfied?	0.76	12.7	61.805	0.71
3. How much are you satisfied with the organizational aspect of your work /how your work is organized?	0.69	11.6	73.424	0.74
4. How much do you think the human rights of the users of your service ward are respected?	0.66	11.1	84.545	0.80
5. How much do you think the human rights of your staff are respected?	0.57	9.6	94.188	0.61
6. How do you evaluate the current state of care in mental health in your service/ward, with reference to resources?	0.34	5.8	100	-0.64
	Eigenvalues	Explained variance		
Extraction sum of the selected factor	2.94	49.0%		

**Table 7a:** Principal component analysis of the WWRR in people with a permanent job (n = 246).

	Total	% of variance	Cumulative %	Component Loading on the Factor
1. How much are you satisfied with your job?	3.44	57.4	57.4	0.75
2. How much do you think the users of your service ward are satisfied?	0.74	12.3	69.8	0.80
3. How much are you satisfied with the organizational aspect of your work /how your work is organized?	0.61	10.1	79.9	0.79
4. How much do you think the human rights of the users of your service ward are respected?	0.49	8.2	88.2	0.83
5. How much do you think the human rights of your staff are respected?	0.41	6.8	95.0	0.66
6. How do you evaluate the current state of care in mental health in your service/ward, with reference to resources?	0.29	4.9	100	-0.68
	Eigenvalues	Explained variance		
Extraction sum of the selected factor	3.45	57.5%		

**Table 7b:** Principal component analysis of the WWRR in people with a fixed-term job (n = 180).

	Total	% of variance	Cumulative %	Component Loading on the Factor
1. How much are you satisfied with your job?	2.74	57.4	57.487	0.65
2. How much do you think the users of your service ward are satisfied?	0.94	12.3	69.821	0.75
3. How much are you satisfied with the organizational aspect of your work /how your work is organized?	0.76	10.1	79.992	0.75
4. How much do you think the human rights of the users of your service ward are respected?	0.66	8.2	88.228	0.75
5. How much do you think the human rights of your staff are respected?	0.55	6.8	95.068	0.45
6. How do you evaluate the current state of care in mental health in your service/ward, with reference to resources?	0.35	4.9	100	-0.65
	Eigenvalues	Explained variance		
Extraction sum of the selected factor	2.74	45.6%		

## APPENDIX

### Scree plot

Principal component analyses with Varimax rotation and Kaiser normalization (which included the inclusion of all components with an Eigen value> 1) were applied.

In the reported scree plots, parallel analysis was added to determine the optimal number of factors and components. In parallel analysis, the scree plot of the observed data is compared with that of a random matrix of the same size as the original. The best solution is based on the number of factors/components with eigenvalues higher than those generated by the random data, both simulated and resampled from the original matrix.
